# Regulation of miR‐200c and miR‐141 by Methylation in Prostate Cancer

**DOI:** 10.1002/pros.23201

**Published:** 2016-05-16

**Authors:** Seodhna M. Lynch, Karla M. O'Neill, Michael M. McKenna, Colum P. Walsh, Declan J. McKenna

**Affiliations:** ^1^ Biomedical Sciences Research InstituteUniversity of UlsterColeraineUK; ^2^ School of MedicineDentistry and Biomedical SciencesQueen's University BelfastBelfastUK; ^3^ Department of Cellular PathologyWestern Health and Social Care TrustAltnagelvin Area HospitalDerryUK

**Keywords:** microRNA, miR‐200c, miR‐141, prostate cancer, DNA methylation

## Abstract

**BACKGROUND:**

In prostate cancer (PCa), abnormal expression of several microRNAs (miRNAs) has been previously reported. Increasing evidence shows that aberrant epigenetic regulation of miRNAs is a contributing factor to their altered expression in cancer. In this study, we investigate whether expression of miR‐200c and miR‐141 in PCa is related to the DNA methylation status of their promoter.

**METHODS:**

PCR analysis of miR‐200c and miR‐141, and CpG methylation analysis of their common promoter, was performed in PCa cell‐lines and in archived prostate biopsy specimens. The biological significance of miR‐200c and miR‐141 expression in prostate cancer cells was assessed by a series of in vitro bioassays and the effect on proposed targets *DNMT3A* and *TET1/TET3* was investigated. The effect on promoter methylation status in cells treated with demethylating agents was also examined.

**RESULTS:**

miR‐200c and miR‐141 are both highly elevated in LNCaP, 22RV1, and DU145 cells, but significantly reduced in PC3 cells. This correlates inversely with the methylation status of the miR‐200c/miR‐141 promoter, which is unmethylated in LNCaP, 22RV1, and DU145 cells, but hypermethylated in PC3. In PC3 cells, miR‐200c and miR‐141 expression is subsequently elevated by treatment with the demethylating drug decitabine (5‐aza‐2′deoxycytidine) and by knockdown of DNA methyltransferase 1 (DNMT1), suggesting their expression is regulated by methylation. Expression of miR‐200c and miR‐141 in prostate biopsy tissue was inversely correlated with methylation in promoter CpG sites closest to the miR‐200c/miR‐141 loci. In vitro, over‐expression of miR‐200c in PC3 cells inhibited growth and clonogenic potential, as well as inducing apoptosis. Expression of the genes *DNMT3A* and *TET1/TET3* were down‐regulated by miR‐200c and miR‐141 respectively. Finally, treatment with the soy isoflavone genistein caused demethylation of the promoter CpG sites closest to the miR‐200c/miR‐141 loci resulting in increased miR‐200c expression.

**CONCLUSIONS:**

Our findings provide evidence that miR‐200c and miR‐141 are under epigenetic regulation in PCa cells. We propose that profiling their expression and methylation status may have potential as a novel biomarker or focus of therapeutic intervention in the diagnosis and prognosis of PCa. *Prostate 76:1146–1159, 2016*. © 2016 The Authors. *The Prostate* published by Wiley Periodicals, Inc.

## INTRODUCTION

miRNAs are small, non‐coding RNA molecules that regulate gene expression by interacting with messenger RNAs (mRNAs), thereby playing crucial roles in fundamental cell processes. In prostate cancer (PCa), several miRNAs are expressed abnormally, suggesting that miRNAs will be useful in the diagnosis, prognosis, and potential therapeutic intervention of this disease 1. It has also become apparent that many miRNAs are controlled by epigenetic mechanisms 2. This is particularly relevant for tumor suppressor miRNAs, which may have become hypermethylated in cancer. Indeed, several tumor suppressor miRNAs have now been shown to be silenced in this manner in a range of tumor types 3, 4, including PCa 5. Thus, epigenetic profiling of miRNAs in PCa may also be useful in terms of both biomarkers and therapeutic targets 6.

The miR‐200 family (hsa‐miR‐200a‐3p, hsa‐miR‐200b‐3p, hsa‐miR‐200c‐3p, hsa‐miR‐141‐3p, and hsa‐miR‐429) is a tumor‐suppressive group of miRNAs that play a key role in suppressing epithelial‐to‐mesenchymal transition (EMT) 7, 8. Demethylation of miR‐200 is also necessary for reprogramming fibroblasts into iPS cells 9. In PCa, a number of reports indicate that expression of the miR‐200 family members is lost, thereby resulting in up‐regulation of targets such as ZEB1, ZEB2, and Slug, which drive EMT and tumor progression 8, 10, 11. However, other studies report that elevated levels of miR‐200 family members are associated with advanced prostate cancer 12, 13, 14, 15. A possible explanation for these conflicting reports is that elevated miR‐200 expression is associated with primary tumors, while loss of miR‐200 expression is found in metastatic cells which have undergone EMT, as others have found is the case in breast cancer 16, 17. If so, the variation observed in the expression of these miRNAs in clinical samples may be a possible way to distinguish indolent from aggressive PCa. However, more research is needed to determine how the expression and regulation of the various family members is influenced at different stages of PCa and EMT progression by other factors.

One such factor which is likely to play a role is methylation, with a number of studies providing evidence that the miR‐200 family is dependent on methylation in several different cancer types 18, 19, 20, 21, 22. Two separate loci are involved; hsa‐miR‐200c‐3p (miR‐200c) and hsa‐miR‐141‐3p (miR‐141) are regulated by the same promoter on chromosome 12, while expression of the other three members is dependent on their shared promoter on chromosome 1 19, 20. Although the miR‐200 family has been firmly implicated as playing a role in PCa, it is surprising that the regulation of their expression by promoter methylation has not been extensively studied in this disease. To our knowledge only one study examining methylation of miR‐200c/miR‐141 promoter in prostate cancer cell‐lines exists 23, and no‐one to date has investigated this specific relationship in PCa clinical tissue. More research is therefore required to determine if the methylation status can help explain the aberrant expression of miR‐200c and miR‐141 reported by other studies in prostate tissue. This is an important consideration because even if miRNAs have potential as biomarkers, analysing those with low expression may be problematic because their detection in either tissue or serum will be difficult. However, if their low expression is due to gene silencing by methylation, their abnormally hyper‐methylated profile acts as a useful proxy positive marker to indicate loss of miRNA expression, a biomarker strategy which has already gained support in studies of *GSTP1* gene methylation in PCa 24. Therefore, in this study we investigated whether the expression of miR‐200c and miR‐141 is related to the DNA methylation status of their promoter in PCa cell lines and then expanded our analyses to prostate biopsy specimens to investigate the clinical relevance of these measurements.

## MATERIALS AND METHODS

### Cell Culture and Transfections

All cell‐lines were obtained from American Type Culture Collection (ATCC). Non‐malignant prostate epithelial cell‐line RWPE1 was cultured in keratinocyte growth medium supplemented with 5 ng/ml human recombinant epidermal growth factor and 0.05 mg/ml bovine pituitary extract (Life Technologies, Paisley, UK). Human prostate cancer cell‐line LNCaP was cultured in RPMI‐1640 supplemented with 10% FBS and l‐glutamine (Life Technologies). Human prostate cancer cell‐lines 22RV1, DU145, and PC3 were cultured in RPMI‐1640 supplemented with 10% FBS and l‐glutamine (Life Technologies). All cells were grown in an incubator with a humidified atmosphere of 95% air and 5% CO_2_ at 37°C and routinely passaged. For miRNA transfections, PC3 cells were seeded at 100,000 cells/well in a 6‐well plate. After 24 hr, cells were transfected with miR‐200c (pre‐miR‐200c), miR‐141 (pre‐miR‐141), or non‐targeting negative control (pre‐neg) (both Life Technologies) at a final concentration of 25 nM using Lipofectamine 2000 (Life Technologies). After 72 hr, cells were harvested for RNA, DNA, or protein extraction. For DNMT1 depletion in PC3 cells, cells were seeded at 100,000 cells/well in a 6‐well plate. After 24 hr, cells were transfected with ON‐TARGETplus SMARTpool DNMT1 siRNA or non‐targeting negative control siRNA (Cont) (both ThermoFisher Scientific, Loughborough, UK) at a final concentration of 100 nM using Lipofectamine 2000 (Life Technologies). After 72 hr, cells were harvested for RNA, DNA, or protein extraction.

### 5‐aza‐2 Deoxycytidine and Genistein Treatment

PC3 cells were seeded at 100,000 cells/well in 6‐well plate. After 24 hr 5‐aza‐2 deoxycytidine (AZA) (Sigma–Aldrich, Poole, UK) was added in fresh media at a final concentration of 1 μM. Treatment was repeated every 24 hr for three consecutive days. After the 3rd day of treatment the cells were allowed to grow for another 24 hr before the cells were harvested for RNA, DNA, or protein extraction. For genistein treatment, PC3 cells were also seeded at 100,000 cells/well in 6‐well plate. After 24 hr genistein (Sigma–Aldrich) was added in fresh media at a final concentration of 40 μM. Treatment was repeated every 24 hr for seven consecutive days. After the 7th day of treatment the cells were allowed to grow for another 24 hr before the cells were harvested for RNA, DNA, or protein extraction.

### Ethics, Consent, and Permissions

FFPE prostate cancer samples were obtained from Altnagelvin Area Hospital, Derry, UK. All patients had provided informed consent for their tissues to be used in subsequent studies. Use of patient material and information, as well as research protocols, were approved by ORECNNI (Ref. 10/NIR02/13). Anonymized patient data for prostatectomy samples is presented in Supplementary Table SI.

### RNA and DNA Extraction From FFPE Human Prostate Tumor Samples

For preparation of RNA and DNA from FFPE needle core biopsies, five 10 μM sections were prepared for each case (n = 14). Following examination by certified pathologist, tumor tissue, and normal adjacent tissue were identified and separated by dissection from the slides, before RNA or DNA extraction was performed. For preparation of RNA and DNA from FFPE prostatectomy biopsy samples (n = 22), five 10 μM sections containing >50% tumor were cut for RNA extraction. Sections of matched normal prostate tissue from the unaffected lobe of the same patient were similarly cut. RNA and DNA extraction on all FFPE tissue was performed using the RecoverAll™ Total Nucleic Acid Isolation Kit for FFPE (Life Technologies) following manufacturer's instructions.

### PCR Analysis

RNA extraction was carried out using Trizol (Life Technologies) according to manufacturer's instructions. 1 μg RNA was used for first strand cDNA synthesis using random primers with transcriptor high‐fidelity cDNA synthesis kit (Roche, Sussex, UK) according to manufacturer's instructions. For quantitative Real‐time PCR (qRT‐PCR), amplification of PCR products was quantified using FastStart SYBR Green Master (Roche) on a Roche LC480 Lightcycler, using primer sets for *SOX2* (Forward 5′‐GGGGGAAAGTAGTTTGCTGCCTCT‐3′ Reverse 5′‐TGCCGCCGCCGATGATTGTT‐3′), *ZEB1* (Forward 5′‐GAACCCGCGGCGCAATAACG‐3′ Reverse 5′‐GCCCTTCCTTTCTGTCATCCTCCCA‐3′), *DNMT3A* (Forward 5′‐AGCCCAAGGTCAAGGAGATT‐3′ 5′‐CAGCAGATGGTGCAGTAGGA‐3′), *TET1* (Forward 5′‐TGGGGTCACTGCTTGCCTGGA‐3′ Reverse 5′‐TCAGTGTTACTCCCTAAGGTTGGCA‐3′), *TET3* (Forward 5′‐CCCCTGAGAGCCCCTTTGC‐3′ Reverse 5‐GTGCCTGCGGATCACCCACTTT‐3′), and housekeeping gene *HPRT* (Forward 5′‐AGCCCTGGCGTCGTGATTAGT‐3′ Reverse 5′‐CCCCTTGAGCACACAGAGGGCTA‐3′). Expression was normalized to *HPRT* and graphs represent the combined results of three independent biological replicates. PCR was also used to confirm knockdown of *DNMT1* (Forward 5′‐GTGGGGGACTGTGTCTCTGT‐3′ Reverse 5′‐TGAAAGCTGCATGTCCTCAC‐3′).

qRT‐PCR of miRNAs was performed using the miRCURY LNA™ microRNA PCR system (Exiqon, Vedbaek, Denmark). A total 50 ng (clinical samples) or 20 ng (cell‐line samples) template RNA was used in each first strand cDNA synthesis reaction. PCR was performed over 40 amplification cycles and fluorescence monitored on the Roche LC480 Lightcycler. For all qRT‐PCR miRNA analysis, normalization was against U6snRNA and graphs represent the combined results from three independent biological replicates, unless otherwise indicated.

### Flow Cytometry

Cell cycle analysis was performed on cells transfected with pre‐miR‐200c, pre‐miR‐141, and negative control, as well as untreated cells. After 72 hr cells were harvested, washed in ice‐cold PBS and fixed in 90% ethanol overnight at 4°C. Following a further wash with ice‐cold PBS, cells were resuspended in 1 ml PBS containing propidium iodide (PI) (10 μg/ml), RNAse A (0.1 mg/ml), FBS (5%), NaN3 (0.02%), NP40 (0.1%), and tri‐sodium citrate (50 μg/ml). After 30 min incubation at room temperature, cells were analyzed on a Gallios™ Flow Cytometer (Beckman‐Coulter, High Wycombe, UK). For apoptotic analysis, transfected cells and controls were harvested after 72 hr treatment and dual‐stained with PI and Alexa® Flour 488‐Annexin V (Life Technologies) following manufacturer's instructions. Stained cells were immediately analyzed on the same Gallios™ Flow Cytometer (Beckman‐Coulter).

### Proliferation and Viability Assays

A cell proliferation XTT assay (Roche) was carried out to measure cell viability. Transfected and control PC3 cells were seeded in a 96‐well plate at 5,000 cells/well and absorbance measured at 24 hr intervals at 490 and 650 nm using a FLUCOstar Omega microplate reader (BMG Labtech, Lougborough, UK). Eight replicates were performed for each experiment and results represent the combined results of three independent biological experiments. For clonogenic assay, treated and control PC3 cells were seeded at 5,000/well in a 6‐well plate and allowed to grow for 1 week. Cells were then washed in PBS, fixed in acetic acid/methanol (7:1 vol/vol) for 5 min and stained with 5% crystal violet for 30 min. Following de‐staining in tap‐water and air‐drying, cell staining was quantified by addition of 1% SDS on a shaker for 30 min to release the dye and measuring absorbance at 595 nm in quadruplicate for each treatment on the microplate reader.

### Bisulfite Treatment

All cell samples were incubated overnight at 55°C in lysis buffer (50 mM Tris pH 8, 0.1 M EDTA, 0.5% SDS, 0.2 mg/ml proteinase K) with rotation, and DNA was subsequently isolated by standard phenol:chloroform extraction. Total DNA of 500 ng was bisulfite converted using the EpiTect Bisulfite Kit (Qiagen, Crawley, UK) according to the manufacturers’ instructions. DNA extraction on all FFPE tissue was performed using the RecoverAll™ Total Nucleic Acid Isolation Kit for FFPE (Life Technologies) following manufacturer's instructions.

### Methylation Analysis

Bisulfite converted DNA was PCR amplified using the PyroMark PCR Kit (Qiagen). Primers were designed using in house PyroMark Assay Design Software 2.0 (Qiagen) and individual forward and reverse primers were obtained from Metabion (Munich, Germany) (Supplementary Table SII). Each reaction was made up to final volume of 25 μl consisting of 2 μl of bisulfite converted DNA, 12.5 μl mastermix, 2.5 μl coral, 1.25 μl of each the forward and reverse 10 μM primers, and 5.5 μl nuclease free H_2_0. PCR amplification was carried on the thermal cycler using the following conditions; initial denaturation step for 15 min at 95°C followed by 45 cycles of 30 sec at 94°C, 30 sec at 56°C, 30 sec at 72°C and a final elongation step for 10 min at 72°C. For COBRA analysis, 1 μg PCR product was added to a restriction digest reaction and made up to a volume of 7.8 μl with nuclease free H_2_0. Digestion was carried out in a final volume of 10 μl containing PCR product, 1X BSA (New England Biolabs, Hitchin, UK), 1X NEB enzyme buffer (New England Biolabs) and 10U of the relevant restriction enzyme (BstU1 or Hinf1). Digestions were carried out at the optimal temperature specific to the enzyme being used for more than 1 hr. The restriction digest reaction volume of 10 μl was separated using agarose gel electrophoresis (3%). For pyrosequencing, samples were run on the PyroMark™ Q24 pyrosequencer and results were analyzed using the associated PyroMark™ Q24 software, v2.0.6 (Qiagen).

For Illumina 450 K analysis, analysis was carried out essentially as before 25. Briefly, genomic DNA was isolated and assessed for purity and integrity as above prior to quantification using the Picogreen fluorescent assay (Life Technologies) as per manufacturer's instructions. In total, 500 ng of high‐quality bisulfite converted (Zymo Research Corporation, CA) DNA was analyzed on the Infinium HumanMethylation450 BeadChip, which was then imaged using an Illumina iScan (Cambridge Genomic Services, Cambridge, UK). Quality Control (filtering out of probes containing of SNPs etc.) and FDR correction was applied to output data prior to the plotting of delta beta values against the genome using GALAXY bioinformatics software 26 and custom workflows. HCT116 WT and HCT116 *DNMT1*/*DNMT3B* double knockout (DKO) DNA was purchased commercially from Zymo Research.

### Statistics

Experiments were carried out at least three times, unless otherwise indicated. Paired or unpaired two‐tailed Student's *t*‐test or two tailed Spearman's rank correlation were used to calculate *P* values where appropriate, with thresholds of ***P < 0.001, **P < 0.01, and *P < 0.05.

## RESULTS

### miR‐200c and miR‐141 Expression in Prostate Cancer Cells and Tissue

**Figure 1:**
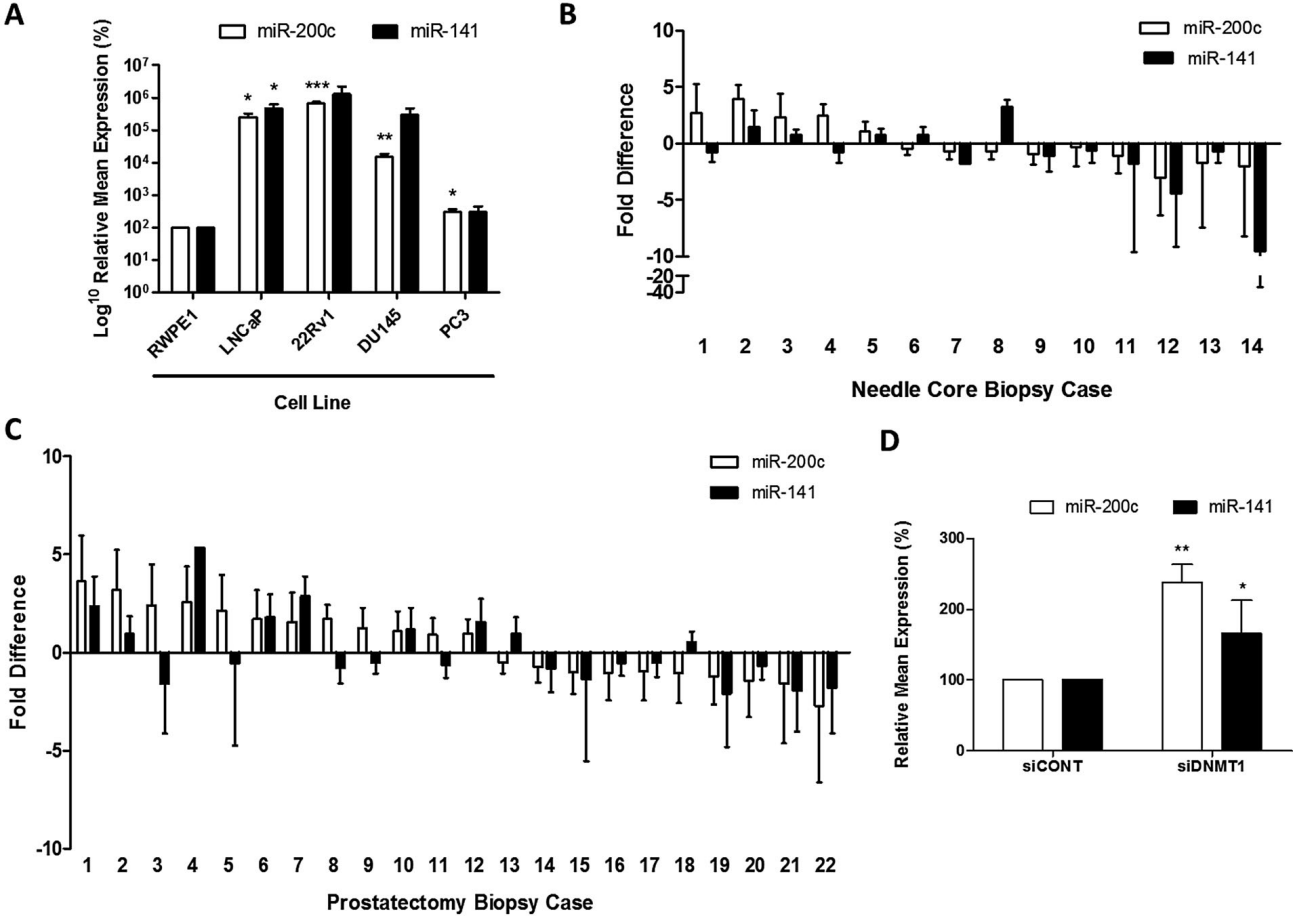
Expression of miR‐200c and miR‐141 in prostate cancer cells and tissues. (**A**) qRT‐PCR analysis of miR‐200c and miR‐141 expression in LNCaP, 22RV1, DU145, and PC3 prostate cancer cell lines and normal prostate epithelial cell line RWPE‐1 (**B**) qRT‐PCR analysis of RNA isolated from needle core biopsy clinical specimens (n = 14) showing fold change expression of miR‐200c and miR‐141 in individual tumor cases relative to matched normal tissue (**C**) qRT‐PCR analysis of RNA isolated from prostatectomy biopsy clinical specimens (n = 22) showing fold change expression of miR‐200c and miR‐141 in individual tumor cases relative to matched normal tissue (**D**) Demethylation treatment by knockdown of DNA Methyltransferase 1 (siDNMT1) in PC3 cells resulted in significantly increased expression of miR‐200c and miR‐141. All data was normalized to housekeeping control U6snRNA and are mean ± SE of triplicate (**A** and **D**) or duplicate (**B** and **C**) experiments. (Student *t*‐test *P* values: *P < 0.05, **P < 0.01, and ***P < 0.001).

miR‐200c and miR‐141 expression was profiled in a panel of prostate cancer cell lines by qPCR. The results from our study show that the expression of both miRNAs is significantly elevated in LNCaP, 22RV1, and DU145 compared to the normal prostate epithelial cell line RWPE1 (Fig. 1A). However, in the PC3 cell line, the miR‐200c expression is markedly decreased compared to the other three cancer cell lines (Fig. 1A). This is interesting because the LNCaP, 22RV1, and DU145 display a predominantly epithelial phenotype whereas the androgen‐independent PC3 cells appear mesenchymal, reinforcing the idea that loss of miR‐200c and miR‐141 expression is associated with increased invasive potential 19, 27. If so, this differential expression may be a crucial way to identify patients with more advanced cancer. In clinical prostate biopsy samples, we observed wide variation in the level of expression of miR‐200c and miR‐141. In both needle core biopsies (Fig. 1B) and prostatectomy tissue (Fig. 1C), up‐regulation as well as down‐regulation of the two miRNAs was observed in tumor tissue compared to paired normal tissue. This suggests that the loss or gain of expression of miR‐200c and miR‐141 across individual patient samples may be indicative of the EMT status of cells within an individual tumor, thereby providing a way to risk stratify patients into low‐ and high‐risk disease categories. This would require more investigation on a larger number of clinical samples to help establish whether miR‐200c/miR‐141 expression will be a useful parameter to help stratify patients into low‐ or high‐risk categories for prostate cancer. Indeed, expression levels of miR‐200c and miR‐141 in a large publicly available data repository of 547 patient samples also show wide variation in prostate tumor samples, compared to normal tissue, where they appear relatively low (Supplementary Fig. S1). This emphasises the value of using a panel of miRNAs, rather than a single marker, if they are to be used for diagnostic or prognostic purposes. It should be noted that although miR‐200c and miR‐141 are co‐expressed, the expression levels in individual samples did not necessarily correlate with each other. This is not unexpected since post‐transcriptional processing means that miRNA family members from the same primary transcript can often be expressed differentially.

### Demethylation Restores miR‐200c and miR‐141 Expression

We were interested to see if the variation observed in expression in our samples correlated with DNA methylation of the miR‐200c/miR‐141 promoter. Since PC3 cells showed the lowest levels of both miRNAs, we investigated if expression of both miR‐200c and miR‐141 could be induced by demethylation. We knocked down DNA Methyltransferase 1 (DNMT1), an enzyme involved in recruitment of methyl groups to the DNA (Supplementary Fig. S2A and B). This results in global DNA demethylation and our results showed that it caused a significant increase in miR‐200c and miR‐141 expression in PC3 cells (Fig. 1D). These results were supported by additional evidence from Illumina 450 K Methylation array data generated from an experiment on hTERT fibroblast cells (Supplementary Fig. S2C and D). This showed that the average methylation of the miR‐200c/miR‐141 promoter in these cells was significantly decreased by knockdown of DNMT1 (siDNMT1) compared to untreated wild type cells (WT). Together, this data indicated that miR‐200c and miR‐141 expression was dependent on methylation in these cells, as we had expected, so we proceeded to design assays to measure methylation in the miR‐200c/miR‐141 promoter region directly.

### Methylation Analysis of miR‐200c/miR‐141 Promoter in Prostate Cancer Cells and Tissues

**Figure 2:**
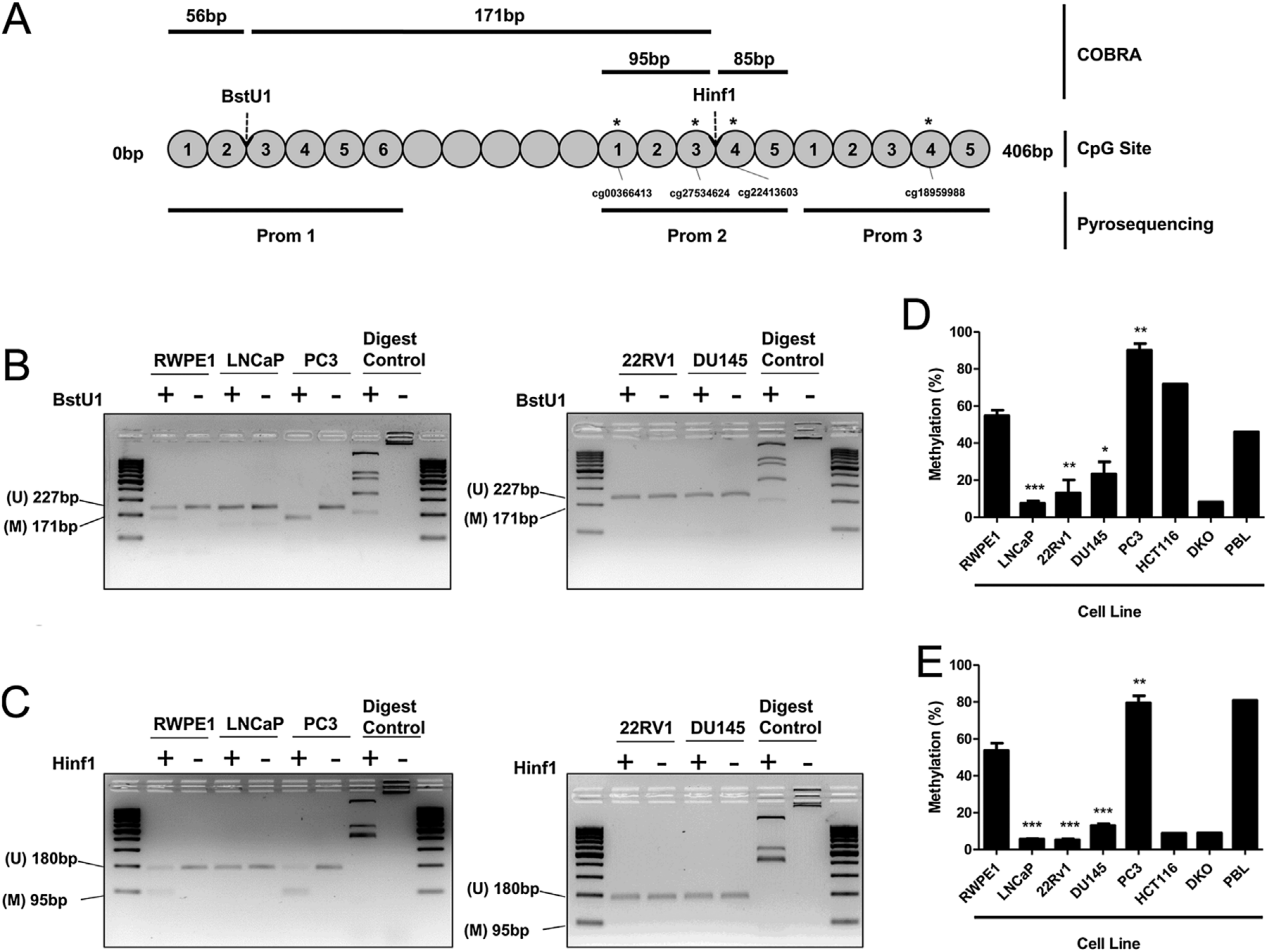
Methylation analysis of miR‐200c/miR‐141 promoter in prostate cancer cell lines. (**A**) Schematic of assay design for COBRA and pyrosequencing to measure methylation at the CpG sites indicated. CpG Sites with asterisks (*) represent those present on the Illumina 450 K array (**B**) COBRA methylation analysis at the BstU1 site and (**C**) Hinf1 site demonstrates no methylation in LNCaP, 22RV1, or DU145 cells at either site. Hypermethylation at both sites in PC3 cells is indicated by digest products of 171 and 95 bp, respectively. (Digest control is miniprep plasmid: +, restriction enzyme added; −, no restriction enzyme added). (**D**) Pyrosequencing of miR‐200c/miR‐141 promoter region 1 (Prom 1) and (**E**) Region 2 and 3 combined (Prom‐2 and ‐3) displays low methylation in LNCaP, 22RV1 cells, and DU145, but significant hypermethylation in PC3 cells relative to normal RWPE1 cells. Control cells (HCT116; colon cancer cell line, DKO; fibroblasts with DNMT1 and DNMT3B knockout, PBL; peripheral blood lymphocytes) show expected results. Images representative of at least three experiments. Data in graphs represents mean ± SE of triplicate experiments. (Student *t*‐test *P* values: *P < 0.05, **P < 0.01, and ***P < 0.001).

Analysis of the miR‐200c/miR‐141 locus revealed a region of 21 CpG sites around the transcriptional start site which we targeted for analysis by Combined Bisulfite Restriction Analysis (COBRA) and pyrosequencing (Fig. 2A and Supplementary Fig. S3). COBRA analysis showed that LNCaP, 22RV1, and DU145 cells were unmethylated in two separate regions, whereas PC3 cells were highly methylated at both (Fig. 2B and C). To quantify this we designed three pyrosequencing primer sets allowing us to accurately quantify the extent of methylation in these two and a 3rd region, altogether covering 16 of the total 21 CpG sites present. These regions were confirmed to have low methylation in LNCaP, 22RV1, and DU145 cells, but were again significantly hypermethylated in PC3 cells (Fig. 2D and E). Given the loss of miR‐200c and miR‐141 expression in PC3 relative to the other cancer cell lines, it appears that this hypermethylation has contributed to the silencing of their expression. These results are in agreement with a previous in vitro study which also demonstrated that DNA methylation correlated with miR‐200c and miR‐141 expression in two of the cell‐lines studied here 23. However, since this has not been explored in clinical prostate tissue, we were interested in examining if this association extended to prostatectomy specimens.

### miR‐200c/miR‐141 Promoter Methylation Status in Prostatectomy Biopsies

**Figure 3:**
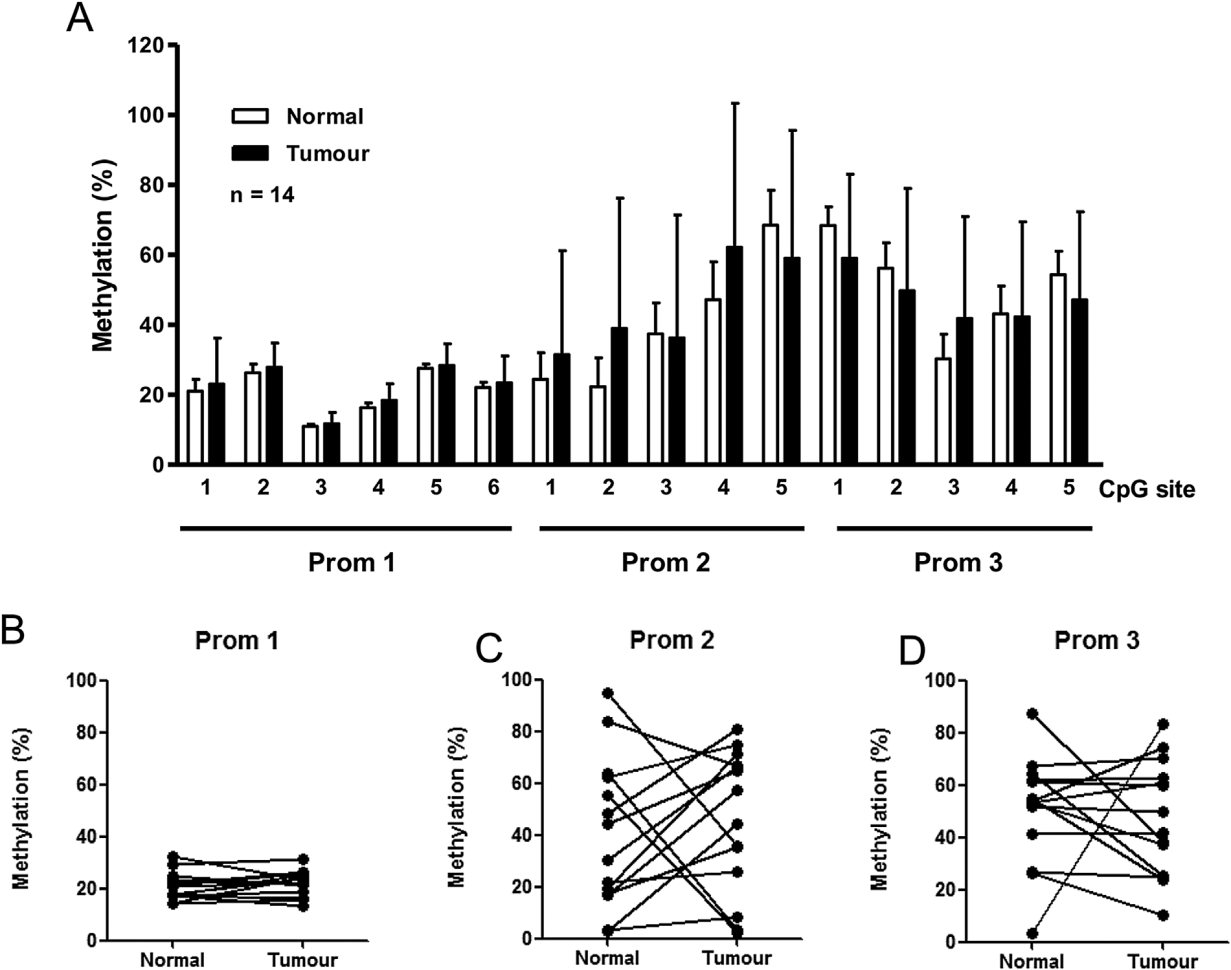
Methylation analysis of miR‐200c/miR‐141 promoter in prostate cancer tissues. (**A**) Pyrosequencing analysis of DNA isolated from prostatectomy biopsy clinical specimens showing average methylation at 16 separate CpG sites within the miR‐200c/miR‐141 promoter in paired tumor samples relative to matched normal tissue (n = 14). Data represents mean ± SE of triplicate experiments. Below, the average methylation in all 14 paired tumor and normal samples is presented separately for (**B**) Prom 1, (**C**) Prom 2, and (**D**) Prom 3 regions of miR‐200c/miR‐141 promoter. Each dot represents the mean methylation percentage of all the pyrosequenced CpG sites in that region.

We were able to successfully extract DNA and quantify the methylation status of all three promoter regions in 14 matched pairs of normal and tumor tissue from clinical FFPE prostate biopsies. Initial analysis of the average methylation in these three regions revealed little difference between tumor and normal samples (Fig. 3A). However, when we viewed each region separately, we noted that normal and tumor tissue showed little variation in CpG site methylation in promoter region 1 (Prom 1), whereas CpG sites in regions 2 (Prom 2), and 3 (Prom 3) displayed much larger variation in methylation (Fig. 3B–D). This raised the possibility that changes at specific CpG sites within the promoter (i.e., those closer to the miRNA loci) may be more important than those further away in determining expression of miR‐200c and miR‐141. Variation in methylation of adjacent CpG sites within the same promoter is not uncommon; for example we had also noted that results from the control cell‐line HCT116 showed marked variation between the three regions with Prom 1 being highly methylated and Prom 2 and 3 having low methylation (Fig. 2D and E). We therefore considered it a prudent step to separately correlate the methylation in these regions with miR‐200c and miR‐141.

### Expression of miR‐200c and miR‐141 Correlates With Methylation at Specific CpG Sites

**Figure 4:**
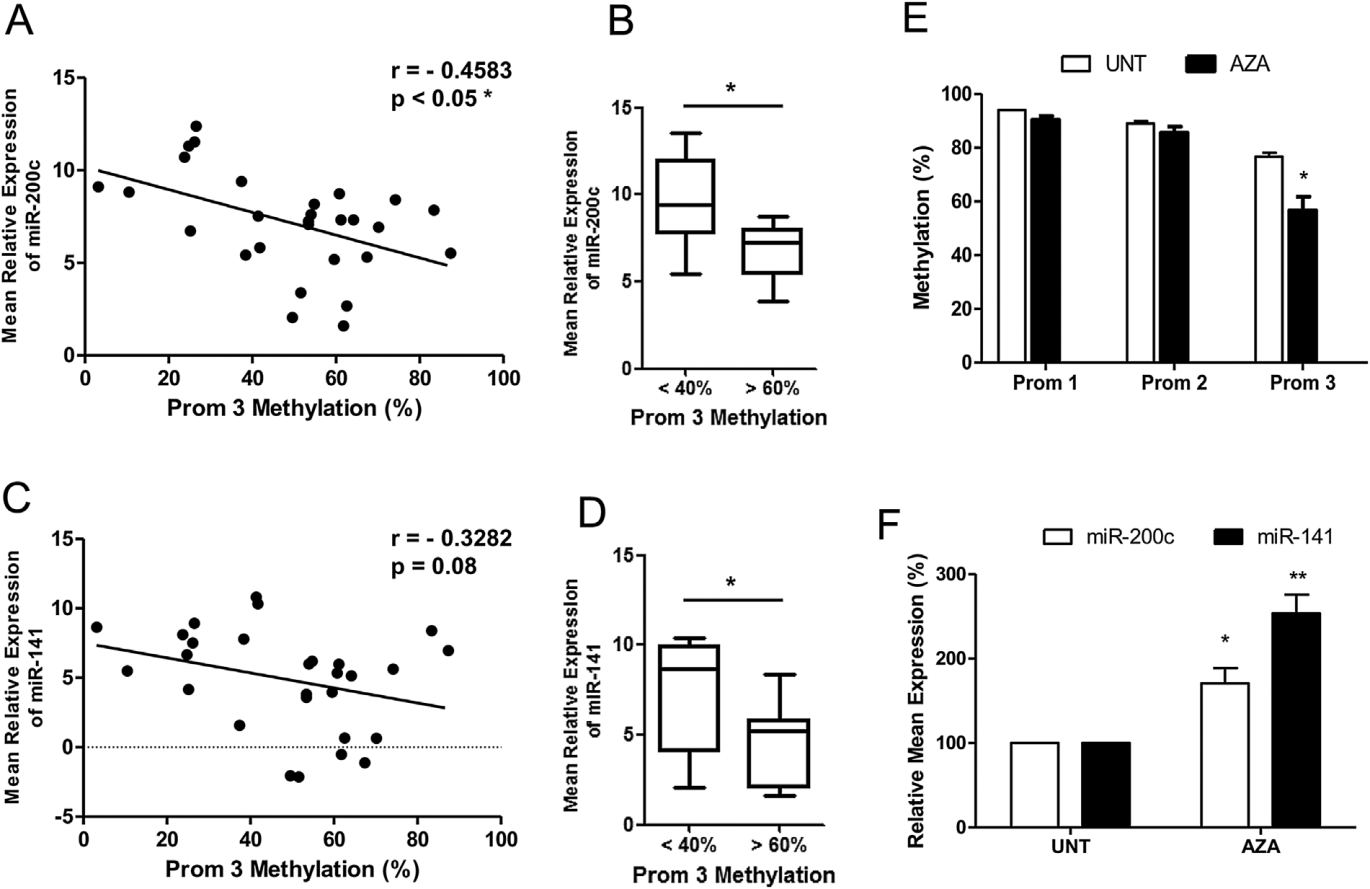
Expression of miR‐200c and miR‐141 correlates with promoter methylation in prostate cancer tissue. Mean expression of (**A**) miR‐200c and (**B**) miR‐141 shows a significant inverse correlation with the mean methylation percentage of the Prom 3 region of the miR‐200c/miR‐141 promoter. (U6snRNA used as internal control, *P* values were generated using Spearman's two‐tailed test: *P < 0.05). Cases with <40% methylation in the Prom 3 region showed significantly higher expression of (**C**) miR‐200c and (**D**) miR‐141 than those with methylation >60% in this region. (*P* values generated using non‐paired *t*‐test with Welch's correction: *P < 0.05). In each box‐plot, median is indicated by horizontal line, whiskers represent 5–95 percentile. (**E**) Treatment with decitabine (AZA) resulted in significant demethylation of CpG sites in the Prom 3 region and (**F**) up‐regulation of miR‐200c and miR‐141. Data in **E** and **F** represents mean ± SE of triplicate experiments. (Student *t*‐test *P* values: *P < 0.05 and **P < 0.01).

When we correlated both miR‐200c and miR‐141 Levels with the methylation status of each promoter region separately, we found that only the Prom 3 region showed a significant inverse correlation with expression (Fig. 4A). Furthermore, samples exhibiting low methylation (<40%) in this region had significantly higher levels of miR‐200c than those with high methylation (>60%). No such correlation was noted when we performed similar analysis for promoter regions one and two separately (data not shown). When we repeated the analysis for miR‐141, we found a similar trend overall (Fig. 4C), with a significant difference in expression between samples showing low and high Prom 3 methylation (Fig. 4D). To confirm the importance of the Prom 3 region, we treated PC3 cells with decitabine (5‐aza‐2 deoxycytidine; AZA) a demethylating agent which is clinically approved for use in the treatment of myelodysplastic syndrome 28. This resulted in significant demethylation of CpG sites in Prom 3, but not those Prom‐1 or ‐2 sites (Fig. 4E). The AZA treatment resulted in significantly increased expression of miR‐200c and miR‐141 (Fig. 4F). Taken together, these results provide evidence that methylation in CpG sites within the proximal region of the miR‐200c/miR‐141 promoter are most important for regulating expression of these miRNAs. This may also be important from a therapeutic standpoint. Several in vitro and in vivo studies have already shown that decitabine can effectively inhibit PCa cell and control tumor growth 29, 30, but have mostly concentrated on pathways related to the androgen receptor (AR). Few have investigated the effect on selected miRNAs and no study to date has investigated if this effect is mediated via the miR‐200 family. Our results suggest that demethylation effects induced by decitabine may contribute to these processes by restoring expression of miR‐200c and miR‐141.

### miR‐200c Inhibits Cell Growth and Increases Apoptosis

**Figure 5:**
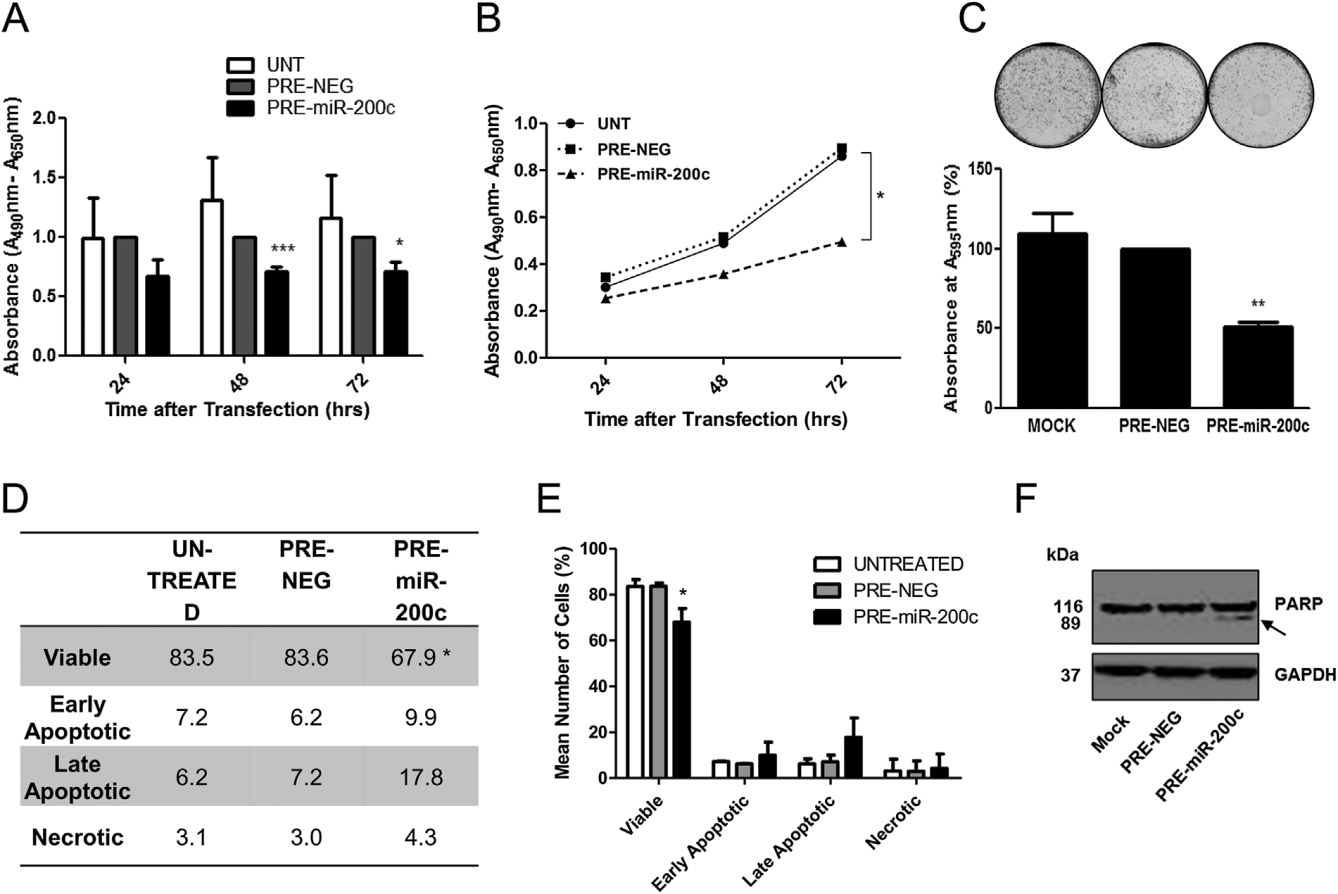
miR‐200c over‐expression inhibits cell growth and induces apoptosis. (**A**) and (**B**) XTT Proliferation assay showing that miR‐200c over‐expression decreases viability of PC3 cells. (**C**) Representative images and quantification of crystal violet staining demonstrates that miR‐200c over‐expression significantly inhibits the colony formation ability of PC3 cells. (**D**) and (**E**) Quantification of Annexin V apoptosis assay showing that miR‐200c over‐expression induces apoptosis in PC3 cells. (**F**) Western blot demonstrating increase of PARP cleavage in miR‐200c transfectants compared to control cells. Cleaved band of 89 kDa indicated by arrow. Images representative of at least three experiments. Data in graphs represents mean ± SE of triplicate experiments. (Student *t*‐test *P* values: *P < 0.05, **P < 0.01).

To assess how epigenetic silencing of miR‐200c and miR‐141 might impact upon prostate cancer cell behavior, we carried out a number of functional bioassays wherein we restored the expression of miR‐200c and miR‐141 separately by transient transfection of PC3 cells. XTT proliferation assays revealed that miR‐200c over‐expression leads to significant inhibition of cell proliferation compared to control cells (Fig. 5A and B). Quantification of crystal violet colony assays indicated that miR‐200c over‐expression resulted in significantly decreased clonogenic capacity (Fig. 5C). In cell cycle analysis we had noted an increase in the Sub G0 fraction of the miR‐200c transfectant cells (data not shown), so we suspected that the apoptotic pathway may be triggered by miR‐200c. Results and representative graphs from an Annexin V flow cytometry apoptosis assay indicated that miR‐200c transfectants exhibited a significant decrease in the number of viable colonies, which may be due to increases in the numbers of apoptotic cells (both early and late apoptotic cells) compared to control transfectants (Fig. 5D and E). Moreover, Western blotting showed an increase in PARP cleavage, indicative of apoptosis within the cells (Fig. 5F, arrow). Together these results provide further evidence that miR‐200c acts primarily in a tumor suppressor fashion, which agrees with previous studies which have examined miR‐200c over‐expression in prostate 10, 11 and other cancers 17, 18, 19, 20, 21, 22. Thus, it seems clear that the loss of miR‐200c expression in tumor cells is likely to promote proliferation and increased invasive potential, due in part to up‐regulation of targets involved in EMT 7, 8. From a patient stratification point of view, it would follow that those individuals exhibiting loss of miR‐200c expression in tumor profiling would be at higher risk of developing invasive PCa. We performed similar bioassays for miR‐141, which also showed significant inhibition of cell proliferation (Supplementary Fig. S4A) compared to control cells. However, the effect on clonogenic potential and apoptosis was less dramatic (Supplementary Fig. S4B–E), suggesting it has less of an influence in mediating this pathway. This may be due to the differential post‐transcriptional processing referred to above or it may need to work in conjunction with miR‐200c (or other miRNAs) to significantly influence cell death signaling.

### miR‐200c and miR‐141 Down‐Regulate DNMT3A and TET1/TET3

**Figure 6:**
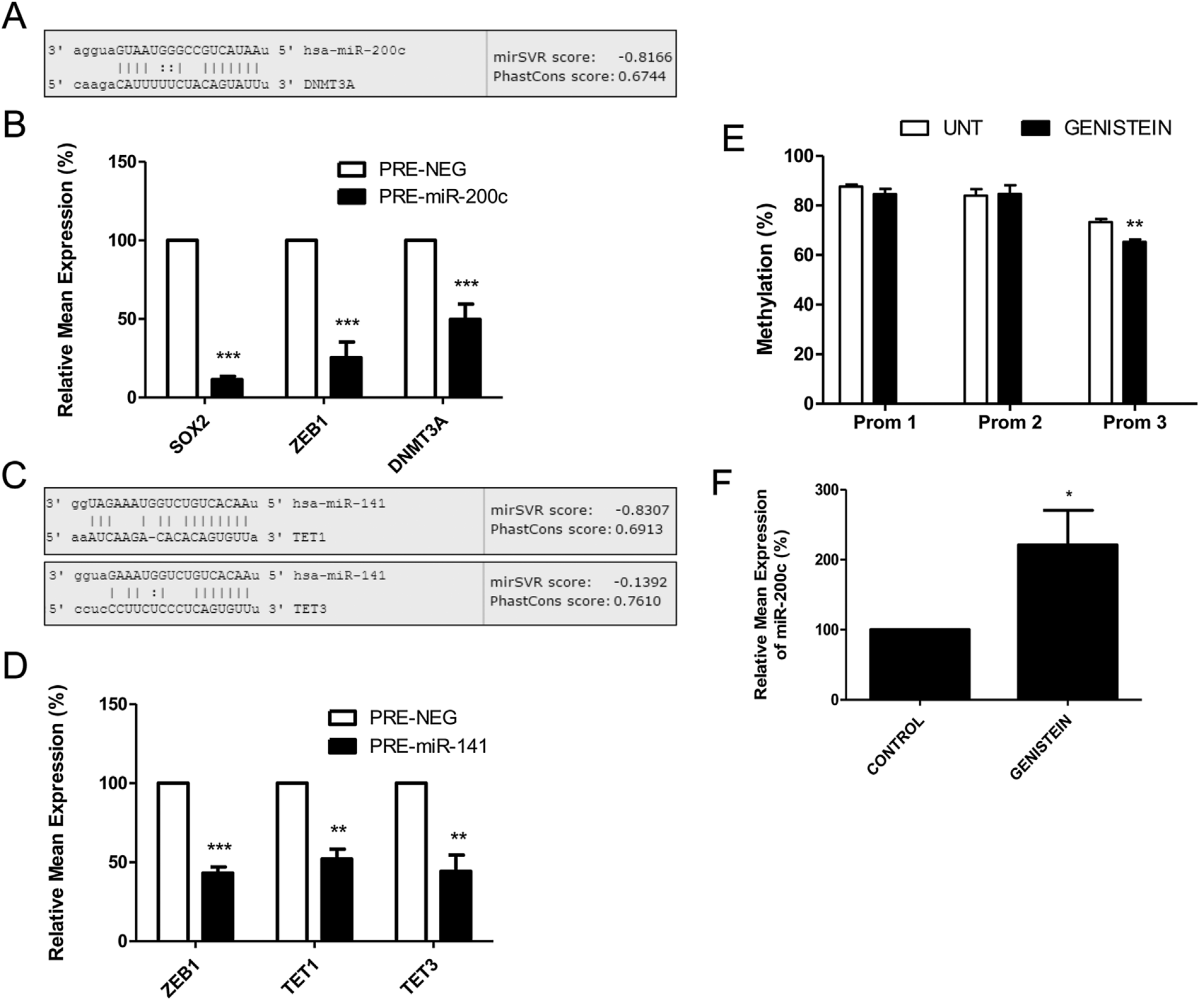
miR‐200c and miR‐141 target genes involved in cell methylation. (**A**) miR‐200c is computationally predicted to target *DNMT3A* by miRanda‐mirSVR analysis (www.microrna.org). (**B**) PCR shows that over‐expression of miR‐200c in PC3 cells results in significant down‐regulation of *DNMT3A*, *SOX2*, and *ZEB1*. (**C**) miR‐141 is computationally predicted to target *TET1* and *TET3* by miRanda‐mirSVR analysis (www.microrna.org). (**D**) PCR shows that over‐expression of miR‐141 in PC3 cells results in significant down‐regulation of *TET1*, *TET3*, and *ZEB1*. (**D**) Pyrosequencing analysis shows treatment of PC3 cells with genistein (40 μM daily for 7 days) results in significant demethylation of miR‐200c/miR‐141 promoter region 3 (Prom 3). Control cells (HCT116; colon cancer cell line, DKO; fibroblasts with DNMT1 and DNMT3B knockout) show expected results (**F**) In the same genistein‐treated PC3 cells, miR‐200c expression is significantly increased as measured by PCR. Data in graphs represents mean ± SE of triplicate experiments. (For all assays treatment compared to controls by student *t*‐test. *P* values: *P < 0.05, **P < 0.01, and ***P < 0.001).

The targets of miR‐200c and miR‐141 that are involved in EMT are well established 7, 8, 18, 19, 20, 21, 22, but in our analysis of online miRNA target databases, we noted with interest that potential targets of both miRNA included components of the cell methylation machinery. miR‐200c was computationally predicted by a number of programs, including the miRanda‐mirSVR algorithm (Fig. 6A), to target DNA Methyl Transferase 3A (*DNMT3A*), which helps regulate DNA methylation. Using similar prediction programs, miR‐141 was predicted to target ten‐eleven translocation methylcytosine dioxygenase genes *TET1* and *TET3* (Fig. 6C), which are important for controlling DNA hydroxymethylation. We proceeded to show that separate over‐expression of miR‐200c and miR‐141 in PC3 cells resulted in a significant down regulation of these predicted target genes (Fig. 6B and D). As controls, known target genes *SOX2* and *ZEB1* were also shown to be down‐regulated as expected. This raises the intriguing prospect that miR‐200c and miR‐141 may also impact upon global methylation as well as EMT. In doing so, they may even contribute to control of their own expression in a negative feedback manner. Significantly, while this manuscript was in preparation, another study also demonstrated that miR‐200c targeted *DNMT3A* in gastric cancer cells and proposed that over‐expressing miR‐200c would result in global DNA hypomethylation and re‐expression of important genes which have been silenced by hypermethylation in this disease 31. A similar approach may be a therapeutic strategy for PCa.

### Treatment With Soy Isoflavone Genistein Restores miR‐200c Expression

The soy isoflavone genistein is an inhibitor of DNMT1 32 and can therefore contribute to the de‐methylation of genes which have been silenced by aberrant hypermethylation. Genistein has been shown to cause increased expression of genes such as *GSTP1*, *RASSF1A*, *BRCA1*, and *BRCA2* by reducing the promoter methylation at these loci 33, 34, 35, so we were interested to see if it might have a similar effect on miR‐200c/miR‐141 promoter methylation and subsequent expression of these miRNAs. Our results show that methylation levels of CpG site in Prom 3 region are indeed reduced after 7 days treatment with 40 μM genistein, with a concomitant increase in miR‐200c expression (Fig. 6E and F). No effect on methylation of CpG site in Prom‐1 or ‐2 region was noted. Although some studies have shown that exposure of cells to genistein alters the expression of various miRNAs, such as miR‐34a 36, miR‐574‐3p 37, and miR‐246a 38, ours is the first study to specifically measure methylation changes in the promoter of the miRNA being investigated. The fact that we see such changes at a low, but physiologically relevant, dose, even after a relatively short treatment period, is noteworthy. Although more study is needed in this area, this provides further evidence for the accepted chemoprotective and anti‐cancer effects of genistein and it is tempting to speculate that this may help explain why populations with high soy diet exhibit lower incidences of prostate cancer 39. Indeed, this is a timely finding, since recent reviews have identified the need for more research into the diet‐epigenome relationship to develop the potential of epigenetic marks as biomarkers of health for use in dietary intervention studies 40, 41. Likewise, reversing the hypermethylated status of genes, including miRNAs, is an attractive strategy from a chemopreventive viewpoint and it has been proposed that the use of dietary interventions would be a possible way to achieve this 42, 43. Our results provide novel evidence to support that theory.

## DISCUSSION

This is the first study to show a correlation between DNA methylation and expression of miR‐200c and miR‐141 in clinical prostate specimens. Moreover, we have shown that methylation in particular CpG sites within the miR‐200c/miR‐141 promoter are more important in regulating expression and we suggest that any future analysis focus on this region. The methylation status in this area of the promoter appears to influence the relative levels of miR‐200c/miR‐141 in prostate tumors, which will subsequently affect the regulation of EMT in the tumor cells. Our data also suggests that aberrant miR‐200c/miR‐141 expression will affect *DNMT3A* and *TET* genes, key components of the methylation machinery which will influence global cellular methylation levels. Finally we show that using either the chemical decitabine or the soy isoflavone genistein can up‐regulate miR‐200c expression in PCa cells by demethylation of specific CpG sites in its promoter. These novel results contribute to our increased understanding of the role of miRNAs in PCa.

It is of course important to acknowledge that the relationship between methylation, expression and functionality of miR‐200c, and miR‐141 in the cell is likely to be mediated through a complex regulatory network (exemplified in Supplementary Fig. S5) and we cannot discount the fact that methylation in other CpG sites, or indeed other epigenetic factors such as histone methylation 18, 23, may also play a role. Nevertheless, the results of this study have increased our understanding of this cross‐talk and may lend themselves to a clinical application. For instance, since our results suggest that miR‐200c acts in a tumor suppressor fashion, it reinforces the idea that any strategy whereby it could be up‐regulated, or re‐expressed, could become a promising approach for the improved treatment of invasive PCa 44.

## CONCLUSIONS

Our findings provide evidence that miR‐200c and miR‐141 are under epigenetic regulation in PCa cells. Profiling their expression and methylation status may therefore have potential as a novel biomarker in the diagnosis and prognosis of PCa. Furthermore, we propose that manipulation of miR‐200c and miR‐141 expression by epigenetic alterations, either through chemical or dietary means, may be the basis for a possible therapeutic intervention for this disease.

## AUTHORS’ CONTRIBUTIONS

SML and DJM carried out the majority of the experimental work and data analysis. KON and CPW provided expertise for the methylation assay design and analyses. CPW gained ethical approval for clinical samples and was involved in the discussion and interpretation of the data. MMM provided expertise for the selection and preparation of clinical samples. DJM, the principal investigator, was responsible for planning, designing, analysis of the data and overall supervision of the work, and final preparation of the manuscript. All authors read and approved the final manuscript.

## Supporting information

Additional supporting information may be found in the online version of this article at the publisher's web‐site.

Supporting Information.
