# Supplementary material for: Regulation of miR‐200c and miR‐141 by Methylation in Prostate Cancer
Source: Prostate. 2016 May 16;76(13):1146–59. doi: 10.1002/pros.23201 (PMC5082568; doi:10.1002/pros.23201)
Supplement: Supplementary file 1 — Supporting Information. [file PROS-76-1146-s001.doc]

**SUPPLEMENTARY INFORMATION**

**Supplementary Table 1. Patient Characteristics**

Clinicopathological data recorded for each of the 22 cases used in the analysis of prostatectomy biopsy specimens.

| **Case** | **Age** | **PSA (ng/ml)** | **Gleason Score** | **TNM** | **High Grade PIN** | **Involves Capsule** | **Circumferential Margin Involved** | **Apical Margin Involved** |
| --- | --- | --- | --- | --- | --- | --- | --- | --- |
| **1** | 65 | 10 | 3 + 3 = 6 | pT2 | Yes | Yes | No | Yes |
| **2** | 57 | 4.5 | 3 + 3 = 6 | pT2 | Yes | No | No | No |
| **3** | 66 | 9.2 | 3 + 3 = 6 | pT2 | Uncertain | No | No | No |
| **4** | 61 | 7 | 4 + 3 = 7 | pT2 | Yes | Yes | No | No |
| **5** | 52 | Not Stated | 3 + 3 = 6 | pT2 | Yes | Yes | No | Yes |
| **6** | 60 | 7.34 | 3 + 4 = 7 | pT2 | Yes | No | No | No |
| **7** | 62 | 10.9 | 3 + 4 = 7 | pT2 | Yes | No | No | Yes |
| **8** | 50 | 13 | 3 + 5 = 8 | pT3 | Yes | No | No | Yes |
| **9** | 67 | 5.7 | 4 + 4 = 8 | pT2 | Yes | No | No | No |
| **10** | 60 | Not Stated | 3 + 3 = 6 | pT2 | No | No | No | No |
| **11** | 53 | 8 | 2 + 3 = 5 | pT2 | Yes | Yes | No | No |
| **12** | 57 | 6.4 | 3 + 3 = 6 | pT2 | Yes | No | Yes | Yes |
| **13** | 41 | 3.9 | 3 + 3 = 6 | pT2 | Yes | Yes | Yes | No |
| **14** | 64 | 9.2 | 3 + 3 = 6 | pT2 | Yes | Yes | Yes | Yes |
| **15** | 62 | 8.3 | 3 + 3 = 6 | pT2+ | Yes | Yes | Yes | Yes |
| **16** | 58 | 10 | 3 + 4 = 7 | pT2 | Yes | Yes | No | No |
| **17** | 47 | 8 | 3 + 3 = 6 | pT2 | Yes | Yes | Yes | Yes |
| **18** | 65 | 6 | 3 + 3 = 6 | pT2 | No | No | No | No |
| **19** | 59 | 5.7 | 3 + 5 = 8 | pT3a | Yes | Yes | No | No |
| **20** | 62 | 17.7 | 3 + 5 = 8 | pT2 | Yes | Yes | No | No |
| **21** | 58 | 4.5 | 3 + 3 = 6 | pT2 | Yes | No | No | No |
| **22** | 62 | 7.2 | 3 + 3 = 6 | pT2 | Uncertain | Yes | Yes | Yes |

**Supplementary Table 2. Primers for Pyrosequencing Assays**

| **Target** | **Primer** | **Sequence** |
| --- | --- | --- |
| miR-200c/miR-141  (Prom 1) | Forward | GGGTTGAGTTTGGGATTGTAGA |
|  | Reverse | AATCCCTAAAAACACTTCCTAATAAAC |
|  | Sequencing | GATGAGGGTGGGTAA |
| miR-200c/miR-141  (Prom 2) | Forward | AGGGTTTGTTTGGATTGTAAT |
|  | Reverse | CCCACCTTAAATCAAACAACTT |
|  | Sequencing | GGTTTGTTTGGATTGTAATT |
| miR-200c/miR-141  (Prom 3) | Forward | AGGGATTTTGGGTTTGAAGT |
|  | Reverse | CACAAAAACAAAAACCTCCATCATTAC |
|  | Sequencing | TGAAGTTGTTTGATTTAAGG |

**
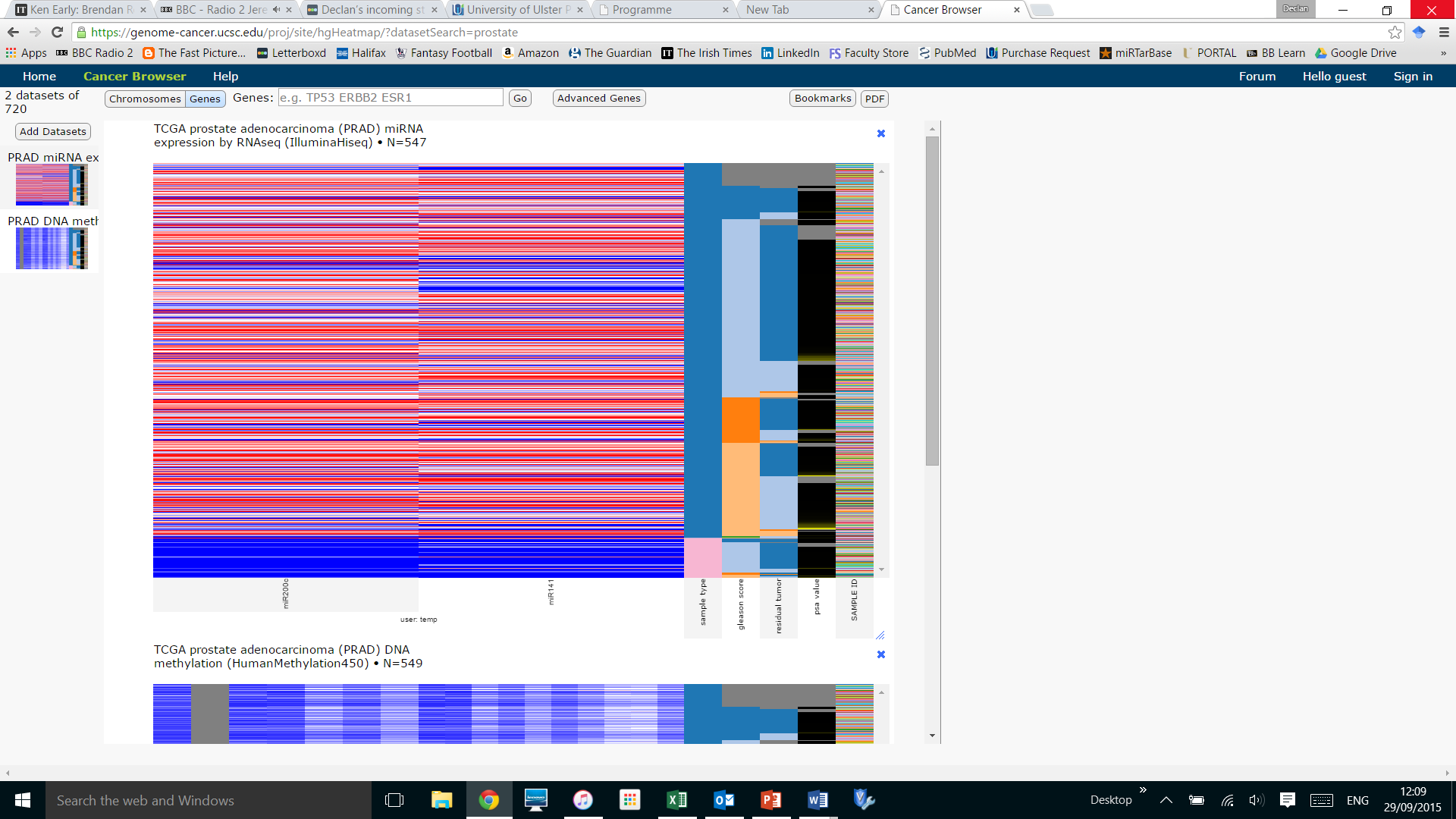
**

**Supplementary Figure 1**

Variable miR-200c and miR-141 expression in a set of 547 patient samples contained in the TCGA prostate adenocarcinoma (PRAD) dataset (ID: TCGA_PRAD_miRNA_HiSeq) publicly available through the UCSC Cancer Genomics Browser (<https://genome-cancer.ucsc.edu/>). The miRNA expression profile was measured experimentally using the Illumina HiSeq 2000 RNA Sequencing platform by the British Columbia Cancer Agency TCGA genome characterization center.


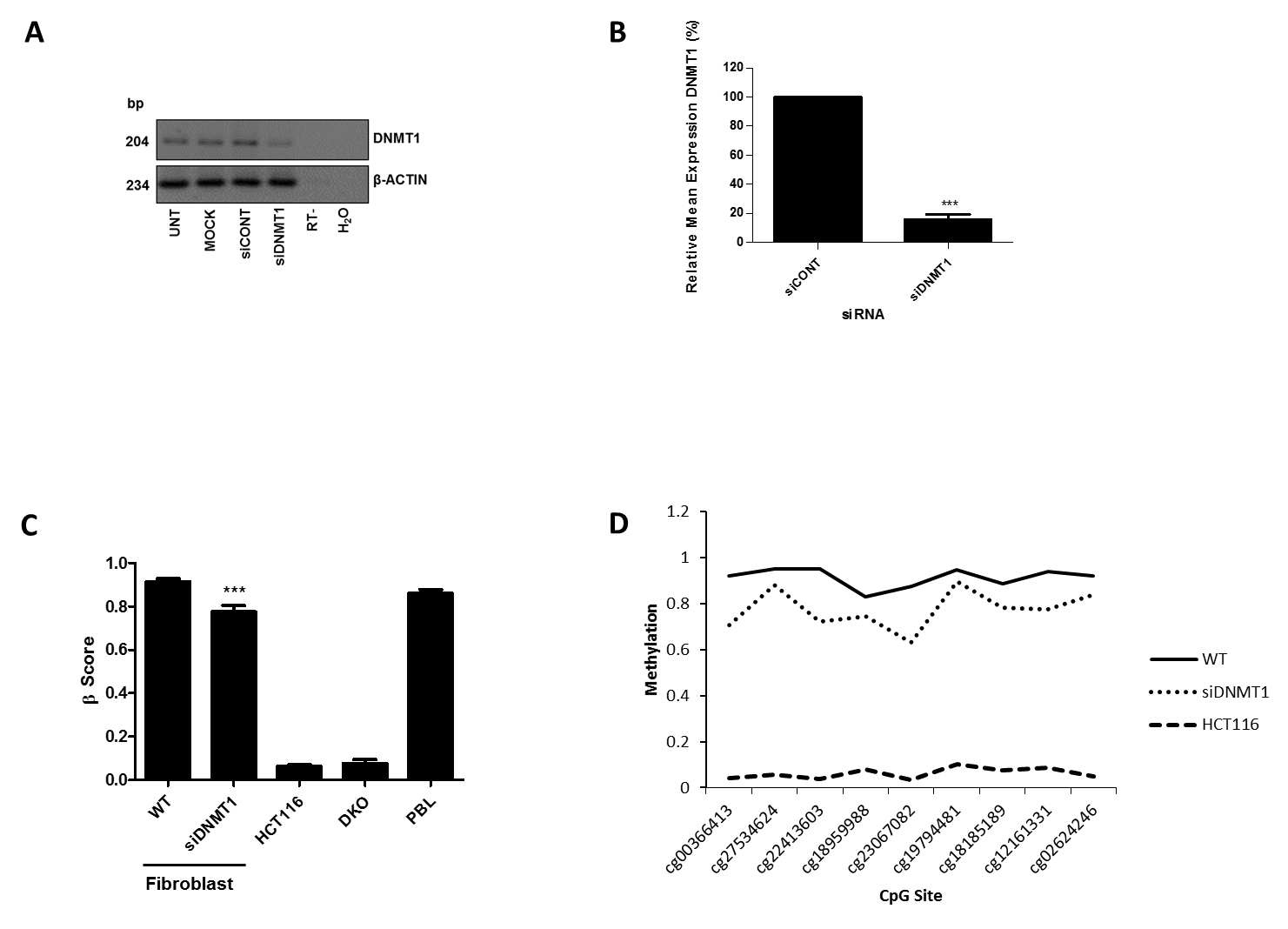


**Supplementary Figure 2**

(A) and (B) DNMT1 knockdown was confirmed by PCR (C) Data from the Illumina 450K Methylation array shows the average methylation (as measured by β score) of the miR-200c/miR-141 promoter in fibroblast cells. Knockdown of DNMT1 (siDNMT1) results in a decrease in methylation compared to untreated wild type cells (WT). HCT116 cells as a control show the expected low methylation levels seen in epithelial cells (D) Individual CpG site analysis from the same Illumina 450K data shows the methylation profile over the assayed region, allowing changes at specific CpG sites to be identified. The largest changes are apparent in the first 5 sites, 4 of which are covered in the pyrosequencing assays. Image representative of at least 3 experiments. Data in graphs represents mean ± SE of triplicate experiments. (Student t-test p-values: *p<0.05, **p<0.01, ***p<0.001)

**Supplementary Figure 3**

Schematic map of region around miR-200c/miR-141 locus showing location of CpG sites. The first 4 CpG sites (cg00366413, cg27534624, cg22413603, cg18959988) are covered in the pyrosequencing assay design (regions indicated by black bars; Prom 1, Prom 2 and Prom 3). For comparison, methylation levels in control cell-lines are also depicted, showing that methylation is higher in the WT hTERT fibroblast line (450K WTK; dark hashes) compared to the HCT116 epithelial line (450K HCT116; light dashes).

**
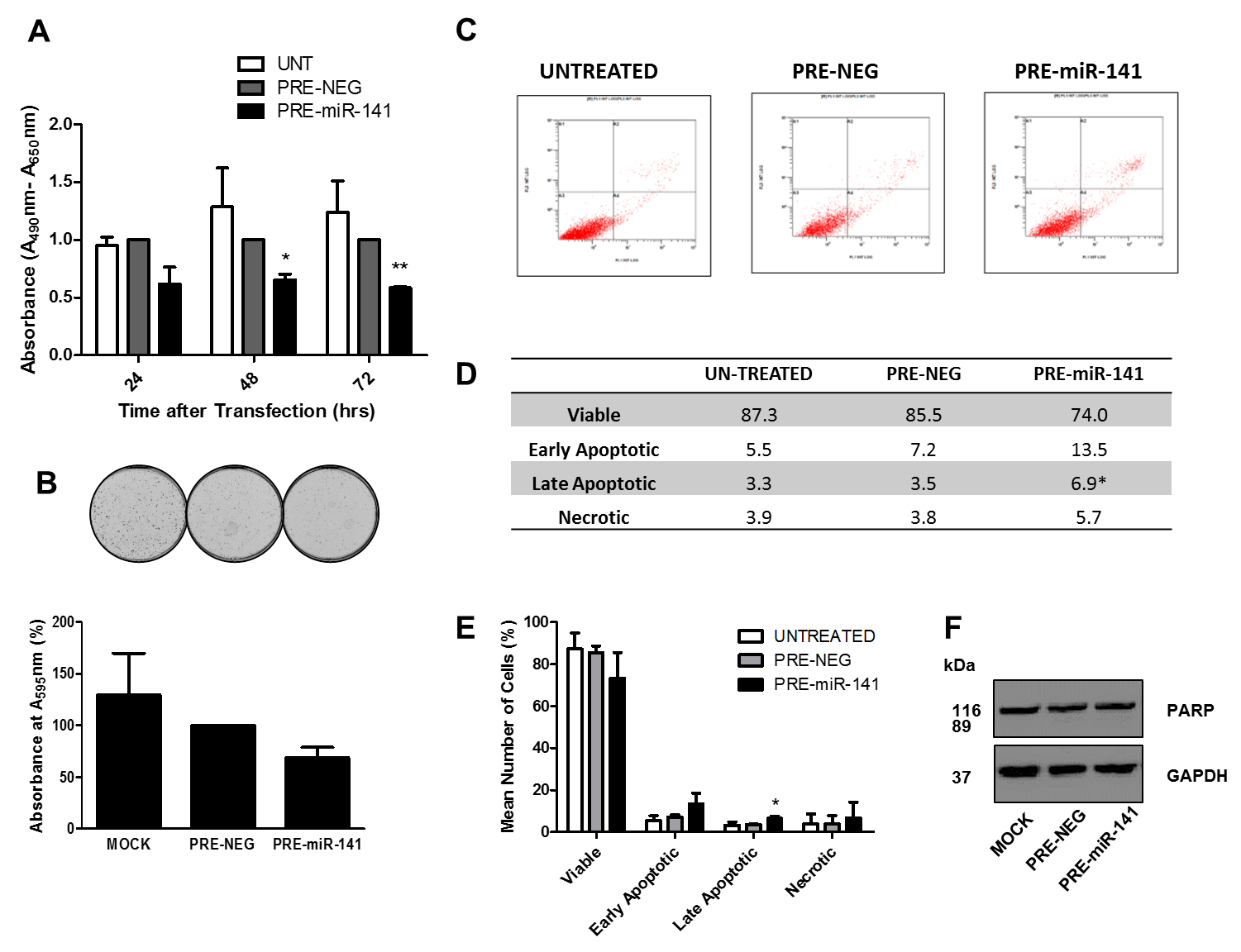
Supplementary Figure 4**

Effect of miR-141 over-expression in PC3 cells (A) XTT Proliferation assay showing that miR-141 over-expression decreases viability of PC3 cells. (B) Representative images and quantification of crystal violet staining demonstrates that miR-141 over-expression shows some inhibition of the colony formation ability of PC3 cells. (C) Representative scatterplots and (D & E) quantification of apoptosis assay showing that miR-141 over-expression induces apoptosis in PC3 cells. (F) Western blot shows limited PARP cleavage in miR-141 transfectants compared to control cells. Images representative of at least 3 experiments. Data in graphs represents mean ± SE of triplicate experiments. (Student t-test p-values: *p<0.05, **p<0.01, ***p<0.001)

**
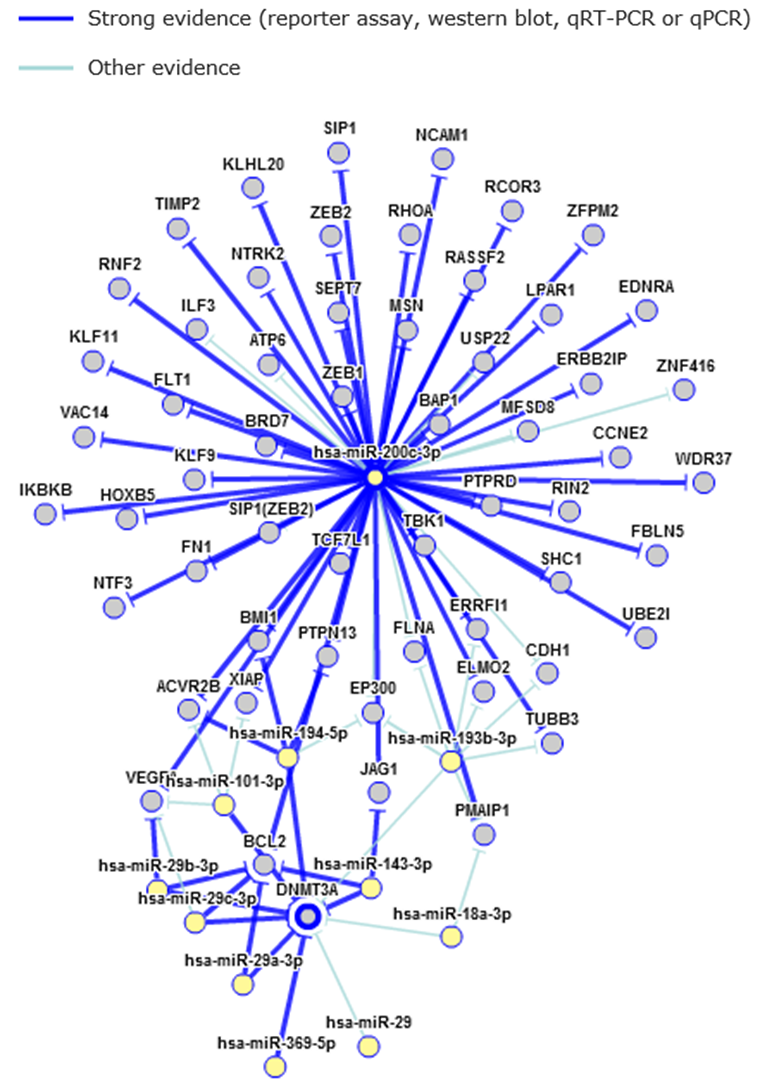
**

**Supplementary Figure 5**

Network analysis of miR-200c – DNMT3A interactions using the miRTarbase website (<http://mirtarbase.mbc.nctu.edu.tw/index.php>) reveals that miR-200c impacts upon several other targets. Likewise, DNMT3A is regulated by several miRNAs. Accession ID: MIRT053571 [miRNA, hsa-miR-200c-3p :: DNMT3A, target gene]
